# Problem formulation for gene drive mosquitoes designed to reduce malaria transmission in Africa: results from four regional consultations 2016–2018

**DOI:** 10.1186/s12936-019-2978-5

**Published:** 2019-10-15

**Authors:** John L. Teem, Aggrey Ambali, Barbara Glover, Jeremy Ouedraogo, Diran Makinde, Andrew Roberts

**Affiliations:** 1 ILSI Research Foundation, 740 Fifteenth Street NW, Suite 600, Washington, DC 20005 USA; 2 NEPAD Agency, Industrialization, Science, Technology and Innovation Hub, 230 15th Road, Midrand, South Africa; 3 ABNE, NEPAD Regional Office West Africa, Hann Maristes 2, Rue HB 350, BP 17204, Dakar, Senegal

**Keywords:** Gene drive, Problem formulation, Risk assessment, *Anopheles gambiae*

## Abstract

**Background:**

Gene drive mosquitoes have been proposed as a possible means to reduce the transmission of malaria in Africa. Because this technology has no prior use-history at this time, environmental risk assessments for gene drive mosquitoes will benefit from problem formulation—an organized and ordered process to identify protection goals and potential pathways to harm to the environment, or animal or human health. Recognizing this need, the New Partnership for Africa’s Development (NEPAD), with support from African and international partners, organized four regional consultative workshops in Africa to initiate this process.

**Methods:**

The workshops were attended by a diverse set of participants and stakeholders, including scientists, ethicists, health professionals, government regulators in the fields of environment health and biosafety as well government policymakers, who met for 4 days to deliberate on protection goals and pathways relevant to the use of gene drive mosquitoes for malaria control. The goal of the workshops was not to produce a comprehensive and detailed environmental risk assessment of gene drive mosquitoes, but rather to introduce problem formulation as a tool to the stakeholder community, and to serve as a starting point for conducting systematic environmental risk assessments in the future, identifying protection goals related to gene drive mosquitoes that are particular to African stakeholders.

**Results:**

Participants in the workshops frequently identified human health and biodiversity as being relevant broad protection goals. Results of the deliberations provide insight into the concerns of African participants at an early stage in the development of gene drive organism/products that should be instructive to developers using this technology.

**Conclusions:**

In general, the African participants of the consultations had a precautionary perspective with regard to environmental risk assessment of gene drive technology. As gene drive technology develops, protection goals will become further refined and candidate products will be further defined. These workshops represent only the beginning of a continuing process that will ultimately inform environmental risk assessment for gene drive mosquitoes to control malaria in Africa.

## Background

Despite intensive control efforts, malaria remains the primary infectious parasitic disease of humans, resulting in 435,000 deaths in 2017 [1]. Although significant progress has been made in reducing the incidence of malaria in recent years, the 2018 World Malaria Report warned that progress had stalled, with about 3 million more cases reported in 2017 (219 million) than in 2016 (216 million). The observation that over 93% of deaths from malaria continued to occur in the WHO African Region in 2017, even though nearly three-quarters (US$ 2.2 billion) of investments in malaria control and elimination efforts were spent there, suggests that new technologies and approaches will be needed. Gene drive technology is being studied as one such novel approach. There are a diverse set of genetic approaches for introducing and spreading genes and traits of interest into and through populations of organisms.

Vector biologists have long appreciated the potential of gene drives as tools for reducing or modifying populations of the world’s deadliest vectors, such as *Anopheles gambiae* and *Aedes aegypti* although, until recently, they have been unable to successfully assemble effective gene drives [2, 3]. With advances in genetic manipulation technology, genetic constructs now can be assembled in the laboratory that when introduced into genomes can be preferentially transmitted to the next generation resulting in the rapid increase in the frequency of the genetic construct in populations of the target species. The system of biased inheritance allowing rapid spread of an introduced trait within a local population is termed gene drive. Numerous strategies have demonstrated the proof of principle for making functional gene drives under laboratory conditions [4–10].

Two general approaches have been proposed for control and elimination of malaria. The first is to introduce gene drives into malaria vectors that will reduce the numbers of vector mosquitoes (population suppression). The second approach is to introduce genes that result in the mosquitoes becoming less competent vectors of the malaria parasite (population modification). Laboratory studies providing proof of principle of both approaches have been reported [7–11]. Both of these approaches are predicted to result in a decrease in malaria transmission [12].

The African Union recently recognized gene drive as an emerging technology with great potential for contributing significantly to Africa’s development [13]. Based on current challenges experienced with vector control interventions to reduce mortality linked to the spread of malaria on the continent, Africa looks to other emerging interventions that can supplement current intervention methods in order to control mosquito populations and or inhibit the transfer of malarial parasites on the continent. The African Union report emphasized that risk assessments will be essential for development of gene drive technologies. It is critical that the capacity and capabilities for making informed decisions about whether to adopt gene drive-based solutions be developed. Therefore, in order to initiate discussions concerning the processes by which environmental risk assessments should be conducted for gene drive mosquitoes, a series of four consultative meetings focusing on building capacity in problem formulation were held in Africa around gene drive technologies. These 4-day workshops, held in Accra, Ghana; Nairobi, Kenya; Gaborone, Botswana; and, Libreville, Gabon during 2016–2018, were organized by the New Partnership for Africa’s Development (NEPAD) Agency. Because many stakeholders will be involved in overseeing the development and use of these technologies, the discussions involved a diverse group of participants representing regional human health and environmental agencies in Africa as well as local and international scientists and other government officials. The workshop participants were chosen based on their involvement with biotechnology, malaria control and scientific technology development in Africa, with no known bias regarding gene drive technology (either in favour or against).

The consultations were intended to (1) acquaint participants with state-of-the-art research on mosquito gene drive technology; (2) familiarize participants with the problem formulation process and its function in environmental risk assessments; and, (3) provide participants with opportunities to consider possible hazards and potential pathways to harm associated with use of gene drive mosquitoes for malaria control in Africa. This manuscript reports on protection goals and hazards that were discussed during these consultations. While this exercise was too brief to provide conclusive results, these initial consultations with participants from countries where malaria is a significant public health burden provide a demonstration of the utility of problem formulation for future risk assessments by developers, regulators and other groups that have an interest in biosafety of gene drive mosquitoes. It is important to recognize that the results of the workshop do not constitute the results of an actual risk assessment for gene drive mosquitoes. Any potential concerns identified by workshop participants should not be interpreted as substantiated risks for gene drive mosquitoes that are supported by evidence from the scientific literature.

### Structure of the workshop

All consultations were preceded by a 1-day pre-workshop session in which participants were offered background information on the biology of *An. gambiae* mosquitoes and use of the CRISPR/Cas-9 gene editing system to engineer gene drive constructs. Presentations ranged from introductory level molecular biology to technical aspects of current gene drive mosquito research and development. The overall structure for the main programme consisted of 2 days of presentations and a day of break-out activities. The initial sessions provided participants with information on relevant biology, including molecular biology and the mechanisms of gene drive, mosquito ecology, regulatory precedents such as biocontrol using exotic species, and stakeholder engagement considerations. Current methodology for reducing *An. gambiae* mosquito populations were reviewed, highlighting the fact that mosquito population suppression is consistent with historical practices to control malaria. Discussions also included the regulatory challenges that transboundary movement of gene drives would present, and how the African Medicines Regulatory Harmonization initiative could serve as a possible model for regulatory harmonization and cooperation within the regional economic communities. This was followed by a presentation on the use of problem formulation to provide scoping for environmental/ecological risk assessment, and lastly, the introduction of four hypothetical case studies (Table 1), which served as the basis for group discussions in break-out groups. The groups did not consider any of the specific gene drive mosquito systems currently being developed by research groups. Each group instead considered one of four different hypothetical gene drive mosquito scenarios exemplifying either population suppression or modification approaches, to allow a more diverse and varied set of possible pathways to harm to be developed.

**Table 1:** Hypothetical case studies

***Case Example 1*** ***Introduction of a novel substance to inhibit*** ***Plasmodium falciparum infection and development*** A novel gene has been introduced to the mosquito genome using the CRISPR/Cas9 system which encodes a protein that inhibits maturation of *Plasmodium* ookinetes. The CRISPR/Cas9 system enables the introduced construct to serve as a template for homology directed recombination, resulting in preferential inheritance of the transgenes by the majority of mosquito progeny. This modification is not primarily intended to alter the behaviour, life cycle or population dynamics of the mosquito, although it may impose some fitness cost (i.e., quantitative reduction in survival or reproduction), but it blocks the successful completion of the malaria parasite life cycle in these mosquitoes, rendering them less likely to transmit the disease to humans.
***Case Example 2*** ***Gene editing of a native gene to inhibit Plasmodium falciparum infection*** A CRISPR/Cas9 gene drive has been engineered for insertion at a site that disrupts proper expression and translation of a gene encoding a cell surface receptor protein that is expressed in the *An. gambiae* midgut. This receptor is required for completion of the *P. falciparum* lifecycle. Although the endogenous function of the receptor is not fully understood, mosquitoes harbouring the mutation show only a modest impact on fitness, but are substantially less able to transmit the disease to humans.
***Case Example 3*** ***Gene editing to affect the sex ratio*** Sex determination in *An. gambiae* makes use of sex-specific chromosomes, where an XX genotype produces a female phenotype and an XY genotype produces a male phenotype. Thus, the sex of the offspring is determined by the chromosome contributed paternally and under natural conditions where the sex ratio is approximately 50/50 male to female. A ‘knock-in’ gene editing construct has been used to insert a novel gene into the *An. gambiae* genome. This novel gene is activated during spermatogenesis and triggers cell death in spermatocytes containing an X chromosome. As a result, male carriers of the gene drive can only sire male offspring. The resulting decrease in the number of females is expected to suppress the population.
***Case Example 4*** ***Gene editing to reduce female fecundity*** A CRISPR/Cas9 gene drive has been engineered to insert at a site that disrupts expression of a protein transporter that is necessary for proper egg provisioning (the transport of materials into developing oocytes from specialized somatic cells). As a result, females carrying the gene drive have greatly reduced fecundity. Males are unaffected and the mating of carrier males with wild-type females is expected to lead to population suppression.

Four hypothetical case studies involving different gene drive mosquitoes were considered by the breakout groups. Two of the case studies involved a modification gene drive designed to render the mosquito population resistant to parasite infection (Case Examples 1 and 2). The other two case studies involved a suppression gene drive designed to reduce the total number of mosquitoes in the target population (Case Examples 3 and 4). It was expected that parasite-sensitive mosquitoes would be progressively replaced by parasite-resistant mosquitoes; yet the total number of mosquitoes in the population would remain unchanged

Protection goals were identified by each group for the particular scenario they considered, and potential hazards and pathways to harm were constructed. To the extent time allowed, the groups then assessed the plausibility of each step in the pathways to harm and considered what additional information would be useful to inform an environmental risk assessment. The results of each break-out group were then summarized and presented to all participants for further discussion.

## Methods

### Problem formulation for environmental risk assessment in break-out groups

#### Identifying relevant broad protection goals

**Fig. 1:**
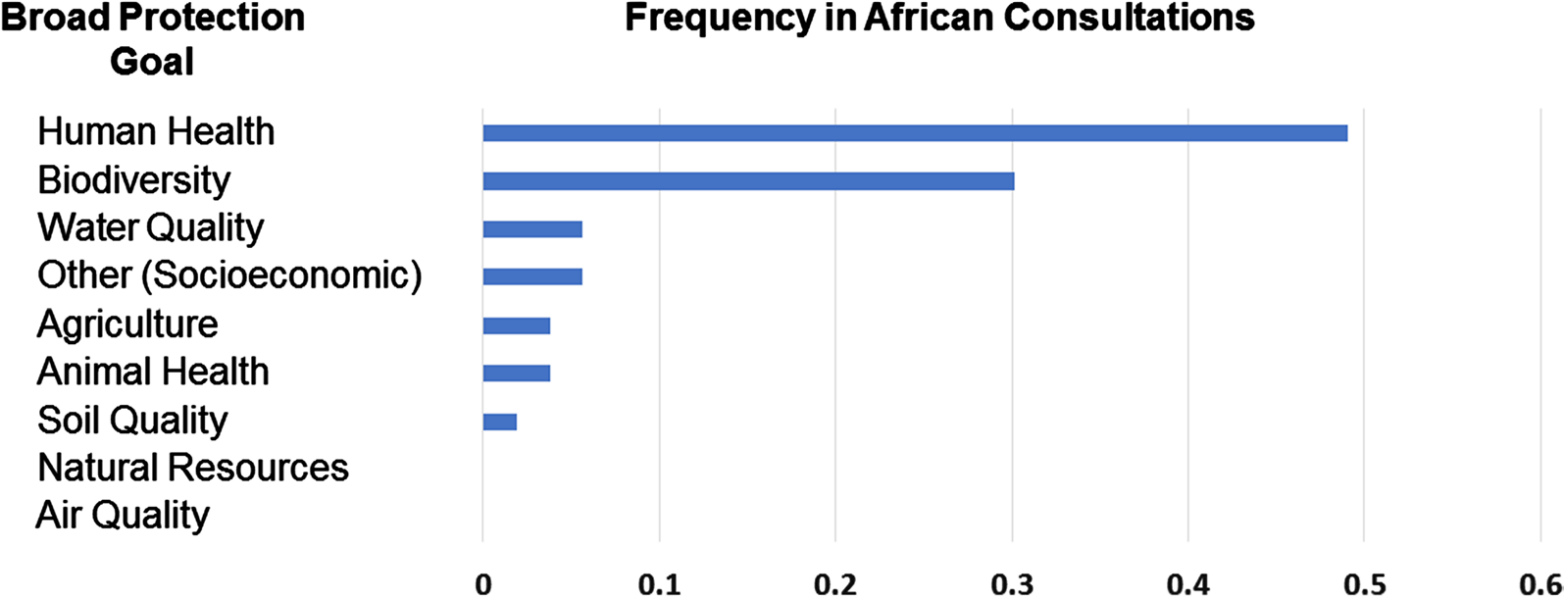
Broad protection goals given to participants as a starting point for discussion. The broad protection goals of human health, biodiversity, water quality, agriculture, animal health, soil quality, natural resources, and air quality are usually defined by statutes in most countries. The relative frequency with which particular protection goals were chosen for further consideration in the construction of pathways to harm is shown in the bar graphs for each protection goal. The bar graph is not meant to assign a ranking of importance of the protection goals, or represent quantitative data produced in the consultations. It is instead an anecdotal assessment intended to convey only a qualitative assessment of the protection goals identified by the participants prior to developing their pathways to harm. The protection goal category ‘Other’ was included to allow participants to create new protection goals not included in the starting point list. For this category, several groups chose socioeconomic protection goals (e.g., valuable services or products important to malaria prevention or cures or that might become less widely available should the introduction of a gene drive mosquito significantly decrease the incidence of malaria)

As a first step in the problem formulation exercise, the participants were asked to identify broad protection goals (Fig. 1). Protection goals correspond to entities of value within the environment, and the purpose of this exercise was to identify those that could possibly be harmed as the result of introducing a gene drive into the local *An. gambiae* mosquito population. Given the relatively short period of time workshop participants were provided with to identify protection goals, their results should not be viewed as definitive, and thus this exercise should be repeated in a more comprehensive manner for guiding future environmental risk assessments. However, it is valuable to note that several patterns emerged, providing insight into the concerns of the various stakeholders present at the four regional workshops. For example, almost every break-out group identified human health and biodiversity as being relevant broad protection goals. Other broad protection goals: soil health, natural resources or air quality were rarely or never identified as relevant protection goals. Goals related to water quality, agriculture and livestock health were identified as relevant by some but not all groups.

#### Elaborating potential pathways to harm

Once break-out groups had identified broad protection goals they considered relevant for environmental risk assessment, they were asked to construct potential ‘pathways to harm’. This is a general tool that helps identify and characterize the ways a particular ‘action’ (in this case the introduction of gene drive mosquitoes) might lead to harmful effects on the broad protection goals [14]. It requires first the refinement of a broad protection goal into a more operational goal, and then the identification of measurable endpoints that could be used to assess harm. The pathway is then constructed in as much detail as possible to identify all the events that must occur between the action and a potential harm. By doing this, it is possible to better identify whether a suggested harm is actually plausible, and if it is, to identify the types of information that will be useful in assessing the likelihood of the harm being realized, and if warranted, plan potential mitigation strategies.

Participants were asked to refine their protection goals, identifying specific entities that could be at risk from the introduction of gene drive mosquitoes into the environment. For example, further refinement of the protection goal ‘biodiversity’ could include identification of a specific harm such as valued predators of mosquitoes that might be affected by the loss of *An. gambiae* within the environment. Having refined their protection goals, the groups were asked to identify a potential pathway to harm. In each case, pathways began with the introduction of gene drive mosquitoes followed by the various consecutive steps that must occur for the harm to be realized. Each step in the pathway was then considered with respect to the likelihood of that step taking place, and ultimately, leading to the identified harm. If a step was deemed to be unlikely, then the pathway to harm was considered implausible. For some pathways to harm there was insufficient information available to judge whether the pathway to harm was plausible or not. In these cases, groups identified additional information that would be useful to make an evaluation of the likelihood that the pathway to harm was valid.

## Results

### Synthesis of the results of the problem formulation exercise

The problem formulation group exercises conducted during the regional consultations were a highly abbreviated form of a standard process that would be followed by regulatory agencies when considering an application for gene drive mosquitoes because the time available in the workshop was limited to only a single day. Moreover, the participants did not have access to additional information from applications or the scientific literature that normally would support decision making. Therefore, while the results described here will be useful for predicting concerns that may arise in environmental risk assessment for future gene drive technologies, they should in no way be considered as conclusive or representative of a consensus perspective.

It is also important to point out that the authors are reporting pathways to harm that were identified in break-out groups without prejudice. Participants in these exercises had various levels of prior experience with the process of environmental risk assessment and its significance/role in regulatory decision making, and for many, the workshop represented the first exposure to the science involved in gene drive technology. This means that, as with all early problem formulation exercises, some of these pathways might not turn out to be plausible based on available evidence, and conversely, other pathways not identified in the early problem formulations may turn out to be significant later. The pathways being reported here, therefore, do not necessarily reflect the views of the authors as to potential risks from the use of gene drive in mosquitoes and are not intended to assert any claim over the likelihood of those harms being realized. Rather, they reflect the types of pathways that were developed by participants in the four consultations and should be useful in improving understanding of what types of harm are relevant for African scientists and regulators at this early stage in the environmental risk assessment process and that may need to be addressed in future environmental risk assessments, either through referencing existing information or by producing relevant data. The protection goals and putative pathways to harm identified in these consultations may also identify areas where additional knowledge would be useful to inform risk assessment and that would need to be addressed from an African regulatory perspective. Because it is not possible to describe fully each pathway developed or elaborated by all 16 of the break-out groups that participated in the four consultations, this synthesis attempts to provide some insight into what sorts of pathways were particularly common and received the most attention from break-out groups as well as listing pathways that may have been unique and possibly not considered in prior exercises of this type, again without prejudice to the likelihood of the identified harm being realized.

**Fig. 2:**
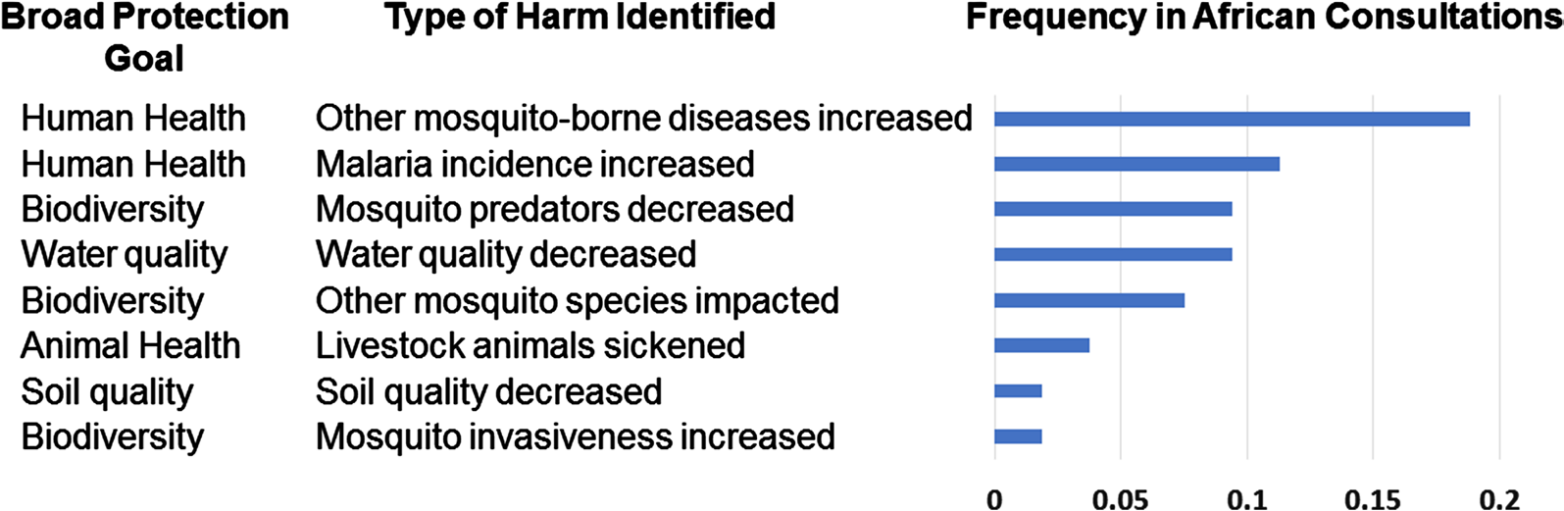
An anecdotal synthesis of common themes identified within the pathways to harm produced in the break-out groups from the four African consultations. As in Fig. 1, the bar graph is not meant to assign a ranking of importance of the themes associated with pathways to harm, or represent quantitative data produced in the consultations. It is instead intended to convey only a qualitative assessment of the various topics pursued by the participants in the course of developing their pathways to harm. Although interpretation should be cautious, it does suggest that certain pathways and related protections goals were of generally greater interest to participants in the consultations

Each group produced at least two pathways to harm that they deemed relevant to their identified protection goals. Human health, biodiversity and water quality were identified repeatedly as protection goals during the four workshops. There were additionally common themes associated with pathways to harm for these protection goals, shown with their relative frequency of occurrence as indicated in Fig. 2. Representative examples of pathways to harm for human health, biodiversity and water quality are shown in Tables 2, 3, and 4.

**Table 2:** Consideration of possible pathways to harm affecting human health

A Protection goal: human health			
Harm: Increase in non‐malarial disease due to other vectors			
Step	Pathway to harm	Plausibility	Additional information needed
1	Introduction of the gene drive mosquito	Given	Literature review, including information/evidence from other control programmes that have eliminated *An. gambiae*^a^
2	Population of *An. gambiae* declines	Likely	
3	Ecological niche opens up providing room for the expansion of another vector population	Likely	
4	Increased possibility of disease transmission	Likely	
5	Other diseases increase (e.g. filariasis, arbovirus, etc.)	Likely	

B Protection goal: human health			
Harm: The modification makes the mosquito capable of transmitting new pathogens, causing new diseases in humans			
Step	Pathway to harm	Plausibility	Additional information needed
1	Introduction of the gene drive mosquito	Given	Capability of mosquitoes to support the development of other pathogens^b^
2	The modification causes unintended effects due to pleiotropy	Unknown	
3	Pleiotropic effect renders mosquito capable of being a vector for new pathogens	Unknown	
4	New/unknown pathogens cause a new disease in humans	Unknown	

A. Suppression Drive, Case Study #3 B. Modification Drive, Case Study #2

^a^[24] provides additional information regarding disease carried by other mosquito species

^b^[25] provides additional information on the evolution of vector competence in *An. gambiae* and mutations affecting parasite virulence [26]

**Table 3:** Consideration of possible pathways to harm affecting biodiversity

A Protection goal: biodiversity			
Harm: Decline in predators of mosquitoes			
Step	Pathway to harm	Plausibility	Additional information needed
1	Introduction of the gene drive mosquito	Given	Literature search: Are there predators that eat only *An. gambiae*?^a^
2	Decline in number of *An. gambiae*	Likely	
3	This leads to decrease in food for predators	Likely	
4	That decrease is not compensated by other food (i.e., other mosquitoes)	Unknown	
5	Decline in predators of mosquito	Unknown	

B Protection goal: biodiversity			
Harm: Enhanced invasiveness of the modified mosquito displaces other species			
Step	Pathway to harm	Plausibility	Additional information needed
1	Introduction of the gene drive mosquito	Given	Literature search: Data on environmental tolerances of modified mosquitoes^b^
2	The gene drive mosquito has increased fitness	Unknown	
3	Other species displaced	Unknown	

A. Suppression Drive, Case Study #3 B. Modification Drive, Case Study #2

^a^[27] provides additional information addressing harm to predators that prey on *An. gambiae*

^b^[28, 27, 29] provide additional information addressing competition between *An. gambiae* and other mosquito species

**Table 4:** Consideration of possible pathways to harm affecting water quality

A Protection goal: water quality			
Harm: Ecosystem/aquatic habitat affected			
Step	Pathway to harm	Plausibility	Additional information needed
1	Introduction of the gene drive [?]mosquito	Given	A large fraction of habitats are temporary and do not support complex communities^a^ No additional information is needed
2	Less larvae breeding in the water	Unlikely	
3	Larvae containing the new gene are present in the water	Unlikely	
4	Aquatic organism populations (e.g., algae and populations (e.g., algae and anaerobic bacteria) increases	Unlikely	
5	Toxin production	Unlikely	
6	Ecosystem/aquatic habitat affected	Unlikely	

B Protection goal: water quality			
Harm: Drinking water becomes contaminated			
Step	Pathway to harm	Plausibility	Additional information needed
1	Introduction of the gene drive Mosquito	Given	Literature review: other vectors of disease, including information/evidence from other control programmes that have eliminated *An. gambiae*^b^
2	Gene spreads into the mosquito population	Likely	
3	Larvae release toxin into the water	Unknown	
4	People drink water	Likely	
5	People get sick (safe drinking water is reduced)	Unknown	

A. Suppression Drive, Case Study #2. B. Modification Drive, Case Study #1

^a^[30] provides additional information addressing larval habitats of *An. gambiae*

^b^[31, 32] provide additional information addressing biosafety of genetically modified organism (genetically modified mosquitoes and genetically modified plants)

Each pathway to harm begins with the introduction of the gene drive mosquito into the environment, followed by a series of steps that must take place in order for harm to occur to a selected protection goal. Workshop participants were instructed to assign a term indicating plausibility to each step in the pathway the harm, selecting from options including ‘likely’, ‘unlikely’ or ‘unknown’. The choice of the plausibility term used by participants was based upon each group’s collective expertise when evaluating the likelihood of each step in the pathway to harm. Due to time constraints, break-out groups did not make use of the scientific literature to evaluate the likelihood term selected. Because the exercise in problem formulation conducted during the workshop did not constitute an actual risk assessment, the designation of plausibility for steps in the pathways to harm, shown for Tables 2, 3, and 4, and the pathways to harm themselves, should not be construed as definitive or representing conclusions regarding risk. The results of the problem formulation activity should instead be viewed as a preview of the concerns held by the African biotechnology community in advance of a formal risk assessment for gene drive mosquitoes.

### Human health

A recurring concern among participants at all the workshops was that the release of gene drive mosquitoes could cause harm to human health by increasing the prevalence of other mosquito-transmitted diseases. Table 2A shows a potentially plausible pathway to harm resulting from the decline in *An. gambiae* caused by a suppression gene drive. As *An. gambiae* infected with the malaria parasite decrease (step 2), an ecological niche becomes vacant, providing room for the expansion of another mosquito vector population (step 3). This leads to an increased possibility of disease transmission from the expanded vector population (step 4). Harm to human health might then result from an increase in the incidence of other mosquito-borne diseases, such as yellow fever or filariasis (step 5). This potential pathway to harm was identified independently in each of the four regional consultations, indicating it is likely to be one of the top concerns about population suppression gene drive strategies that will be raised by regulators and other stakeholders. However, across the four workshops, there was lack of consensus about the likelihood that each step in the pathway would occur. Some cited support for the pathway steps in the scientific literature, while others expressed a need for more information regarding the potential for mosquitoes to compete for an ecological niche and considered the plausibility of step 3 to be “uncertain”. Given the frequency with which this pathway to harm appeared during the consultations, it would be valuable to collect available scientific data regarding the concept of a niche for *An. gambiae* (and also a niche for mosquito-borne pathogens) to provide a context for risk assessors to address this question in the future.

Increased incidence of other mosquito-borne diseases was also frequently identified as a pathway to harm to human health for population modification gene drives (Table 2B). In this example, it was postulated the genetic modification would cause unintended effects due to pleiotropy (step 2). As a consequence of the pleiotropic effects, vector competence of the mosquito would increase for parasites that are normally not carried by *An. gambiae* (step 3). Harm to human health would then occur in the form of new diseases transmitted to humans by *An. gambiae* (step 4). The specific steps involved in altering the vector competence of the mosquito as a result of genetic modification could not be readily defined by this group, so the plausibility of this step in the pathway was deemed ‘unknown’, requiring additional information. Information from the scientific literature regarding changes to vector competence as a result of mutation in the mosquito would be useful to risk assessors in assessing the plausibility of this pathway to harm.

Like the example in Table 2B, other pathways to harm were also identified in modification scenarios that were the consequence of mutations arising in the gene drive mosquito. For example, another pathway to harm raised the possibility that an increased rate of mutation within mosquitoes could lead to increased fitness over time resulting in a “super fit” mosquito with an increased capacity to transmit malaria. In another pathway to harm it was suggested that the mutation of the gene drive mosquito could result in a longer lifespan, increasing the incidence of malaria transmission. For these pathways to harm involving the evolution of new traits over time, uncertainty was high on one or more steps in the pathway, and it was anticipated that additional information would be needed to determine the likelihood that these steps could occur.

Other pathways to harm affecting human health were based on the idea that toxic or allergenic substances could be produced in the gene drive mosquito, and that these substances could be transmitted to humans either directly by biting, or indirectly by exposure from substances released into the environment. In some cases, the potential for toxicity was perceived to be related to the components of the gene drive itself and in others, it was suggested to result from ongoing mutagenesis within the mosquito genome leading to the production of toxic or allergenic proteins. Participants indicated that there was uncertainty as to the steps leading to the generation of toxic or allergenic proteins and suggested that a review of the scientific literature would be needed to further assess the plausibility of these types of events.

Another frequently identified pathway to harm affecting human health involved harm in the form of an increase in the incidence of malaria (Fig. 2). The principal version of this pathway to harm involved mutation of the *Plasmodium falciparum* parasite in response to the population modification mechanism in the mosquitoes in a way that would increase its capacity for infection of the gene drive mosquitoes, possibly leading to a higher incidence of human infections. Only those break-out groups considering population modification case studies identified this pathway. There was uncertainty regarding the likelihood that mutation could alter the properties of the parasite in this way, thus more information from the scientific literature would be required to assess the validity of this pathway to harm. It was also mentioned that malaria could increase by a different route, in which success of gene drive mosquitoes in transiently eliminating malaria exposure would cause human populations to lose their low-level immunity [15] over time. In this scenario, harm to human health would follow in the form of a higher disease burden upon a resurgence of malaria at a later time.

### Biodiversity

The second most common broad protection goal considered relevant in almost every break-out group was biodiversity. Groups found it more challenging to develop and refine operational protection goals and measurement endpoints, simply because of the huge scope of biodiversity. However, participants generally focused on a few areas of biodiversity that might be directly impacted by the release of gene drive mosquitoes.

Table 3A exemplifies a potential pathway to harm affecting predators of *An. gambiae* that results from the introduction of a gene drive mosquito designed to suppress mosquito populations. Following the introduction of the gene drive, there is a decrease in the number of mosquitoes available as prey for predators (step 2), and predators are unable to compensate by feeding on alternative food sources (step 3). Some participants suggested specific predator examples, including leopard frogs and jumping spiders. As a consequence of reduced *An. gambiae* mosquitoes as prey, predators of mosquitoes decline (step 5). There was uncertainty associated with the last two steps of this pathway to harm because participants did not know whether there are any predator species that rely on *An. gambiae* exclusively as a food source and suggested that further research in the scientific literature would be required. Indeed, a recent publication indicates that this pathway to harm is unlikely, as a literature review revealed no species that rely solely on *An. gambiae* as prey [16].

Another proposed pathway to harm affecting biodiversity reflected concern that mutations in the mosquito could lead to a competitive advantage of the gene drive mosquito as compared to wild-type, causing increased invasiveness and leading to the displacement of other mosquito species (Table 3B). In this pathway to harm, the genetic modification results in a mosquito with increased fitness (step 2). Over time, the gene drive mosquito displaces other species as a result of its fitness advantage (step 3). Pathways to harm involving invasiveness were limited to population modification drive case studies.

### Water quality

Water quality was initially considered a protection goal by many break-out groups because the immature stage of mosquitoes is aquatic. However, only a few groups actually developed pathways to the harm affecting water quality, and steps within those pathways were often subject to high levels of uncertainty. Table 4A shows a proposed pathway to harm affecting water quality that results from the introduction of a gene drive mosquito designed to suppress populations. In this pathway to harm, mosquito larvae in the aquatic environment decline as a result of the gene drive (step 2), resulting in reduced larval consumption of algae (step 3) causing levels of algae to increase (step 4). Toxins produced from algal bloom (step 5) result in negative effects on wildlife in the aquatic habitat (step 6). In this potential pathway to harm, steps 2 through 6 were considered unlikely, hence the pathway to harm was determined to be implausible (no additional research is needed).

Other potential pathways to harm affecting water quality identified in break-out groups were based on the notion that there could be toxic substances produced by gene drive mosquito larvae that could be released into the aquatic environment (Table 4B). In this case, the pathway to harm involved the spread of the gene encoding the toxic substances within the population (step 2) and the subsequent release of toxin into the water (step 3). People consuming the water (step 4) were sickened (step 5), resulting in reduction of water for human consumption (step 6). The plausibility of the pathway was determined to be ‘unknown’ as it could not be determined from the hypothetical case studies whether toxin production was possible.

Other protection goals that occurred less frequently in the African consultations included animal health and soil quality (Fig. 1). These were occasionally identified as relevant protection goals at the outset of the break-out groups, but participants rarely chose them for elaboration of pathways to harm. However, one group constructed a pathway to harm resulting in an increase in Rift Valley Fever, a livestock disease that is borne primarily by *Aedes* and *Culex* mosquitoes. The pathway to harm was virtually identical to the example described in Table 2A (where other disease vectors increase as a result of suppression of *An. gambiae* and other species of mosquito fill the vacant niche), but in this case with the harm manifested in animal livestock rather than humans. The group considered this pathway to harm to be “plausible”, but recognized a need for further investigation of the scientific literature. Harm to soil was considered a possibility if mosquitoes expressed a toxic protein and it entered the soil as a result of mosquitoes dying and falling to the ground. As with other pathways to harm involving the notion of toxic proteins, the participants expressed uncertainty with regard to the potential for gene drive component proteins to be toxic (or have the potential to generate toxic proteins from other genes within the genome).

### Comparing the results of the African consultation to previous problem formulation exercises for gene drive mosquitoes

Many of the same pathways to harm identified in the African consultations were also identified previously in a problem formulation workshop in Reston, Virginia in 2016 [17]. All the same themes represented in the African consultations (Fig. 2) were also represented in the Reston workshop. However, there were some themes that were unique to the African consultations. For example, participants in the African consultations were more inclined to consider how the behaviour of mosquitoes could be negatively impacted by genetic modification. They anticipated that changes in mating behaviour could possibly result from genetic modification, affecting interactions with other *An. gambiae* mosquitoes or non-target organisms. They postulated that changes in behaviour could additionally affect nectar-feeding behaviour on plants (and consequently, pollination) such that the reproduction of medicinal plants could be affected. They further considered that genetic modification might alter the taste of the mosquito, changing its attractiveness to predators and possibly affecting the mosquito population size. It was further suggested that changes in behaviour as a result of the genetic modification of the mosquito could also affect competition between mosquitoes at different points in the life cycle, as both larvae and as adults, and this could also affect population size at the corresponding life stages.

Although water quality was identified as a protection goal in both the Reston workshop and the African consultations, there was greater concern amongst the African consultation participants that aquatic habitats would be negatively affected by gene drive mosquito larvae. Participants had concerns that consumption of genetically engineered mosquito eggs/larvae in untreated drinking water could be harmful, either to fish in the aquatic environment or to people drinking the affected water (as in Table 4B). The release of toxins into the water was additionally speculated to be a possible cause of skin irritation in humans. It should be emphasized that these concerns are not the result of a rigorous risk assessment, nor have they been examined with respect to validity based upon the scientific literature. They are presented here simply to show the extent to which participants in the Reston workshop and the African consultations shared or differed in their concerns regarding commonly identified protection goals.

## Discussion

Gene drive modifications of malaria vector mosquitoes are being developed with the goal of future use in Africa within the next decade. These workshops provided an initial opportunity for African stakeholders to hear about the current state of research on gene drive technologies for malaria control and to understand the role of problem formulation and risk assessment in considering the acceptability of new technologies. They further provided an opportunity for participants to consider ways to construct a regulatory system that is tied to national or regional protection goals. As such, these workshops represented an important engagement effort that allowed for exchange of perspectives and insights into local, national and regional concerns that should inform and improve the development of these technologies.

Although differences were observed in specific pathways to harm and in participants’ characterization of likelihood or uncertainty for individual steps, the African consultations identified very similar protection goals and pathways to harm as those generated in a previous problem formulation workshop held in Reston, Virginia in 2016. This suggests that there are unlikely to be important pathways to harm that were missed because of the composition of the participants or the location of the workshop. It also suggests that future risk assessments of gene drive mosquitoes in Africa will be able to make effective use of problem formulation to identify risks associated with gene drive mosquitoes as the gene drive technology approaches practical application.

Problem formulation is an important step in identifying the types of information that will be useful in conducting environmental or ecological risk assessments. Participants first identified environmental protection goals applicable to their regions and subsequently considered four hypothetical cases describing different types of gene drive systems in *An. gambiae*, a prominent malaria vector. For each case, they developed potential pathways to harm affecting two relevant protection goals that were pertinent to their interests and assessed whether additional information was needed to determine the validity and (when possible) the plausibility of the pathway. Although this problem formulation exercise was focused primarily on risks that gene drive mosquitoes might pose to human health or the environment, social and ethical concerns were also widely discussed among the participants. These discussions, undertaken at the early stages in the development of gene drive technology, will be important for incorporating legal, social and scientific context into the planning for environmental risk assessment of applications for gene drive-based malaria control in the future [18, 19], and for identifying needs and gaps per local, national and regional regulatory requirements.

### The relevance of biological control organisms as a framework for considering the use of gene drives in *Anopheles gambiae*

Classical biological control has been used to successfully control a variety of insect pest species in Africa [20–22], and involves the intentional release into the environment of non-native insects that will spread and persist. It is understood that once released, insects used for biological control cannot be recalled and that permanent changes to the environment may result from such an action. Prior to release, biological control agents are thoroughly evaluated with regard to risks that they may pose to the environment and problem formulation is an integral part of the process. Although there are significant differences in the risk assessment of a classical biocontrol agent as compared to a gene-drive organism (one being that host specificity is the main factor for consideration in biocontrol risk), it is useful to recognize that a precedent exists for risk assessment and release of organisms that result in a lasting change to the environment.

### The prominence of social and communications issues as a component of the discussions

Inclusion of socio-economic issues in environmental risk assessment associated with gene drives has been recommended by the US National Academy of Sciences of Engineering and Medicine and others [18, 19]. Although the social and ethical aspects of using gene drive technology to control mosquito vector populations were not the intended focus of the consultation, these issues were widely discussed among participants and were recognized as an important component of decision making around the use of gene drive technology. It was recognized that efforts to listen and learn from public opinions through the early stages of project development and beyond will be essential to the success of any gene drive-based vector control programmes. Evidence for the importance of public support in this process was provided by the example of the Eliminate Dengue programme, which made a significant investment in public engagement prior to releasing male *Aedes aegypti* mosquitoes infected with *Wolbachia* bacteria as a means of reducing dengue virus transmission [23]. As a result of concerted efforts to maintain good communications with community partners, the programme was able to garner public support for the programme that was essential to its success. Although the practical aspects of using a gene drive mosquito were not discussed in detail, the participants discussed the need for regional harmonization of regulatory frameworks as a necessary prerequisite for addressing issues of transboundary movement. Similarly, participants were not asked to consider issues beyond pathways to harm and potential harms to the environment. However, during the course of discussion participants discussed the need to consider international obligations, such as to those under the Cartagena Protocol on Biosafety and related to issues, such as liability and redress.

### The importance of social aspects of the technology in informing policymakers

The ethics of utilizing gene drive technology to target mosquitoes within the environment was addressed briefly within the consultations in Africa. There was a general consensus among the participants that reducing or eliminating mosquitoes for the benefit of human health was, at least hypothetically, an acceptable use of gene drive technology, and that this was consistent with historical practices to control malaria. Notably, many of the participants attending the consultations had acquired malaria one or more times as a consequence of living and working in Africa, underscoring the importance of conducting these environmental risk assessment exercises in problem formulation in the actual locations where the gene drive mosquitoes would ultimately be used by participants who are intimately familiar with the disease. The need to consider the cultural context of using gene drive technology in Africa will additionally be important, as some participants felt that guidance from religious leaders would be an important criterion for determining the suitability of the technology within their communities.

### Perspectives on problem formulation

When surveyed regarding the usefulness of the problem formulation workshops, most participants were in agreement that the activity was valuable and that it is important to consider environmental risk assessment for gene drive mosquitoes at the present time, while the technology is still in the development stage. In general, the African participants of the consultations had a precautionary perspective with regard to environmental risk assessment of gene drive technology. Although willing to rule out potential harms based upon evidence from the scientific literature, they often doubted the sufficiency of existing scientific information to make a determination of the plausibility of harm, favouring the collection of additional data through experimentation. This perspective gave them flexibility to conceive a wide range of pathways to harm involving the potential unintended effects of a genetically modified mosquito, even if those pathways were discarded or not elaborated upon further consideration. As gene drive technology develops and candidate products are further defined, the relevant protection goals will be further refined. These workshops thus represent only the beginning of a continuing process that will ultimately inform environmental risk assessment for gene drive mosquitoes to control malaria in Africa.

## Conclusion

The elimination of malaria has been a longstanding goal for human health programmes, and would not only reduce human mortality but also provide tremendous socio-economic benefit through increased productivity and quality of life for populations living in malaria-endemic regions. However, if this goal is to be realized, new technologies will likely need to be applied in conjunction with existing efforts. One technology that has the potential to contribute to the elimination of malaria is the use of gene drives to introduce desirable traits into vector mosquito populations in order to reduce malaria transmission. These technologies will be subject to risk assessment in order to ensure that they do not have the potential to cause undue harm to the environment, ecology or human health. Prior to the release of any such technology, it is important to hold conversations to help define the areas of concern that will likely need to be addressed in those risk assessments.

The series of African regional consultations reported here represents a first step in fostering this dialogue. African scientists and regulators, identified by NEPAD, were invited to receive information about the underlying science behind the use of gene drives for vector control, and then to participate in a series of exercises intended to identify and define the potential areas of concern. The results of these exercises demonstrate broad commonality among African scientists and regulators in terms of their concerns over the use of gene drive vector mosquitoes, which are generally well aligned with conclusions generated in similar activities conducted elsewhere. Participants in these consultations were generally conservative and precautionary, and it is important to note that the pathways to harm developed during the workshop relied on participants’ personal knowledge and the information presented during the course of the workshop. As such, the results are not intended to reflect a risk assessment for any future uses of gene drive technology. Instead, these outcomes serve to identify the areas of concern that developers and regulators will likely need to address before any use of gene drives in vector mosquitoes as a component of malaria control programmes.

## Data Availability

Not applicable

## Abbreviation

NEPAD: New Partnership for Africa’s Development
